# Er-intercalated Ti_3_C_2_T_*x*_ MXene electrocatalyst for efficient energy conversion

**DOI:** 10.1039/d5ra05111h

**Published:** 2025-10-08

**Authors:** Shamaila Fatima, Irfan Ali, Aumber Abbas, Azhar Ali Haidry, Syed Rizwan

**Affiliations:** a Physics Characterization and Simulations Lab (PCSL), Department of Physics & Astronomy, School of Natural Sciences (SNS), National University of Sciences and Technology (NUST) Islamabad 44000 Pakistan syedrizwan@sns.nust.edu.pk syedrizwanh83@gmail.com +92 51 886 5599; b School of Materials Engineering, Jiangsu University of Technology Changzhou 213001 China; c College of Materials Science and Technology, Nanjing University of Aeronautics and Astronautics 211100 Nanjing PR China; d Department of Physics, University of Okara Okara 56300 Pakistan

## Abstract

For sustainable green hydrogen production, bifunctional catalysts must rival or surpass precious metal electrocatalysts in water splitting. MXenes, with their rich surface chemistry, unique physicochemical properties, and stability, have emerged as promising candidates. However, achieving balanced hydrogen evolution (HER) and oxygen evolution (OER) activity in a single medium remains challenging. Herein, we report the synthesis of Ti_3_C_2_T_*x*_ MXene and erbium intercalated (Er@Ti_3_C_2_T_*x*_) nanocomposites as bifunctional electrocatalysts for overall water splitting in alkaline media. The Er@Ti_3_C_2_T_*x*_ catalyst demonstrates outstanding HER performance, requiring only 256 mV overpotential at 10 mA cm^−2^ with a Tafel slope of 102 mV dec^−1^, while also exhibiting superior OER activity with an overpotential of 381 mV at 10 mA cm^−2^ and a Tafel slope of 157 mV dec^−1^. Electrochemical tests were conducted in 1 M KOH using an Ag/AgCl reference electrode and a Pt wire as the counter electrode. Chronoamperometry confirmed long-term stability and durability. Structural and morphological analyses conducted using XRD, SEM, EDX, FTIR, and Raman spectroscopy verified the successful intercalation of Er while preserving the 2D MXene structure. A notable increase in *d*-spacing from 8.9 Å (pristine MXene) to 12.2 Å (Er@Ti_3_C_2_T_*x*_) further confirmed erbium (Er) incorporation. Moreover, electrochemical impedance spectroscopy (EIS) revealed reduced charge-transfer resistance, highlighting enhanced kinetics and efficiency for water-splitting reactions.

## Abstract 2

For sustainable green hydrogen production, bifunctional catalysts must rival or surpass precious metal electrocatalysts in water splitting.

## Graphical abstract

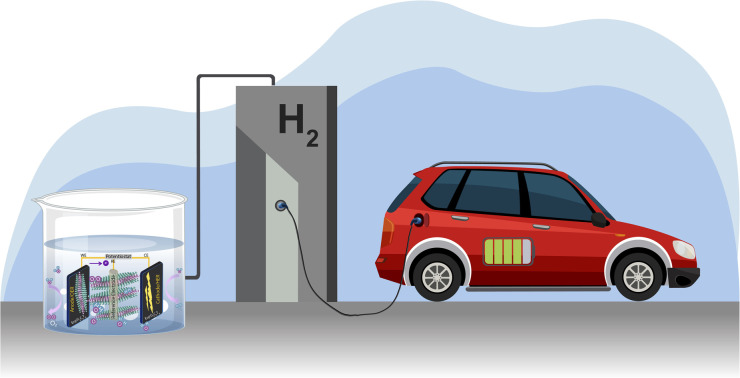

## 1 Introduction

The considerable increase in energy demand, driven by population growth and improved living standards, poses a major global challenge. It is estimated that the energy demand will increase by nearly 50% by 2030.^1,2^ Most developing countries still rely on conventional fossil fuels,^3^ which release carbon dioxide and other toxic by-products, threatening human health and the environment. To address this, renewable energy resources such as solar, wind, and water have gained widespread attention.^4^ Among them, hydrogen has emerged as a clean and sustainable energy carrier, with hydrogen combustion offering environmentally friendly energy conversion due to its zero carbon emissions.^5^ As the global demand for clean energy escalates, the development of efficient, scalable, and cost-effective hydrogen production methods has become imperative^6,7^

Electrochemical water splitting^8,9^ is one of the most promising green strategies for hydrogen generation,^10^ producing hydrogen and oxygen gases through the application of electrical current. Different electrolysis technologies have been developed, including Solid Oxide Electrolysis Cells (SOEC),^11^ Alkaline Water Electrolysis (AWE),^12^ Anionic Exchange Membranes (AEMs)^13^ and Proton Exchange Membranes (PEMs).^14^ The efficiency of these processes depends strongly on the performance of electrocatalysts. Precious metals such as Pt and Ir remain benchmark catalysts for HER and OER, respectively, but their scarcity and high cost hinder large-scale applications.^15,16^ Consequently, research has shifted toward earth-abundant alternatives, such as transition metal chalcogenides,^17,18^ graphene,^19^ graphene-based composites,^20^ metal–organic frameworks (MOFs),^21^ and MXene composites^22^

MXenes, first reported by Gogotsi and colleagues in 2011,^23^ are a new class of 2D transition metal carbides, nitrides, and carbonitrides with the general formula M_*n*+1_X_*n*_T_*x*_, where M is an early transition metal (like Ti, V, Mo, Nb, Cr, *etc.*), X is C and/or N, T_*x*_ denotes surface terminations (–O, –OH, –F, –Cl) and *n* varies from (1–4). More than 70 MXene compositions have been synthesized experimentally, while over 100 are predicted theoretically. MXenes have transitioned from the early synthesized forms that had just one or two metal atoms to the latest high-entropy 2D MXenes that incorporate three metal atoms (Ti_3_SiC_2_, Ti_3_AlC_2_)^24^ selectively removing the A-layers from the corresponding ternary carbide or nitride MAX phases. Compared to rGO (reduced graphene oxide)^25,26^ and copper,^27^ MXenes exhibit high conductivity, hydrophilicity, chemical stability, large surface area, and tunable surface chemistry, making them attractive for energy-related applications, including water splitting, batteries,^28^ and supercapacitors.^29^ For HER, the metallic conductivity of MXenes facilitates fast charge transfer, while theoretical studies suggest that M_2_X and M_3_X_2_ can provide active sites for hydrogen adsorption.^30^ However, the catalytic activity is strongly influenced by surface terminations, as –F and –O groups can impede ion diffusion or alter adsorption energetics.^31,32^

To enhance MXene activity, various modification strategies have been reported.^33,34^ Among them, doping or intercalation with rare-earth metals is particularly effective in tuning surface chemistry and electronic properties.^35–37^ Rare-earth elements can reduce surface –F/–OH groups, improve charge transfer, and introduce new active sites.^38^ Specifically, Er doping has been shown to modify MXene surface functionalities, leading to improved hydrogen adsorption and catalytic activity. The –F and –OH terminations have been observed to be reduced by erbium doping in an MXene.^39^

In this study, we present the first-ever synthesis and electrochemical evaluation of Er-intercalated Ti_3_C_2_T_*x*_ (Er@Ti_3_C_2_T_*x*_) MXene as a bifunctional electrocatalyst for overall water splitting in alkaline media. Unlike previous reports that mainly focused on either HER or OER activity of rare-earth-modified MXenes, our work emphasizes dual catalytic performances, long-term stability, and structural reinforcement achieved through Er intercalation. The Er atoms were incorporated into the MXene layers *via* a simple van der Waals-mediated self-assembly process, which, unlike conventional methods requiring harsh post-treatments, offers a facile, template-free, and solution-based route with controllable Er loading while preserving the intrinsic 2D lattice. The intercalation of Er effectively pillars the interlayer galleries, expands the *d*-spacing, introduces new catalytic centers, and reduces charge-transfer resistance, thereby accelerating reaction kinetics. Among the tested compositions, the optimized Er@Ti_3_C_2_T_*x*_ (0.5 : 2), denoted as T_1_, exhibited the most favorable catalytic performance for both the anodic (OER) and cathodic (HER) reactions, significantly outperforming pristine Ti_3_C_2_T_*x*_. Furthermore, the optimized catalyst demonstrated remarkable stability in alkaline media, highlighting its promise as a cost-effective and durable alternative to precious-metal-based electrocatalysts.

## 2 Experimental section

Materials such as Ti_3_SiC_2_ MAX powder (300 mesh size, 95% pure), hydrofluoric acid (HF 48 wt% in H_2_O ≥ 99%), TMAOH (trimethyl ammonium hydroxide 25%), potassium hydroxide (KOH), *N*-methyl-2-pyrrolidinone (NMP), absolute ethanol, deionized water (DI H_2_O wt% purity 99%), erbium nitrate hexahydrate (Er(NO_3_)_3_·6H_2_O) and 5% concentration of Nafion (Binder) were utilized in synthesis. All materials and chemicals were employed exactly as received from the supplier (Sigma-Aldrich) and without any prior modification.

**Fig. 1:**
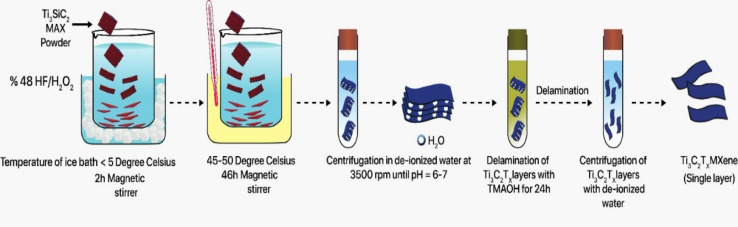
Synthesis process of Ti_3_C_2_T_*x*_ (etching & delamination).

Fig. 1 illustrates the synthesis process for producing multilayered Ti_3_C_2_T_*x*_ MXene *via* wet chemical etching, followed by the preparation of erbium-doped Ti_3_C_2_T_*x*_ MXene through a sonication treatment as explained below.

### 2.1 Ti_3_C_2_T_*x*_ MXene synthesis

In a Teflon beaker, 5 mL H_2_O_2_ and 45 mL hydrofluoric acid (HF) were mixed for the etching of Ti_3_SiC_2_ MAX. The HF/H_2_O_2_ mixture was stirred in an ice bath for 30 minutes before adding 3 g of Ti_3_SiC_2_ MAX. The ice bath must be kept at 5 °C since the etching process is exothermic and releases H_2_ gas (Fig. 1). The bottle's lid must be kept unfastened to allow the gas to escape. The solution-filled Teflon beaker was placed at 30 °C in an oil bath for 45 hours for the selective etching of Si layers of the MAX phase. After the completion of the reaction, the received product (MXene) was washed 5 times in 45 mL centrifuge tubes. Finally, Ti_3_C_2_T_*x*_ MXene was collected after a successful washing procedure and placed in a vacuum oven to dry for 24 hours at a temperature of 80 °C.

The chemical reaction is as follows, where eqn (2) and (3) lead to different surface terminations, and the reaction occurred with HF as an etchant.

1 Ti_3_SiC_2_ (s) + 3HF (aq) = Ti_3_C_2_ (s) + SiF_3_ (aq) + 3/2H_2_ (g)

2 Ti_3_C_2_ (s) + 2HF (aq) = Ti_3_C_2_F_2_ (s) + H_2_ (g)

3 Ti_3_C_2_ (s) + 2H_2_O (aq) = Ti_3_C_2_ (OH)_2_ (s) + H_2_ (g)

### 2.2 Ti_3_C_2_T_*x*_ MXene delamination

To delaminate Ti_3_C_2_T_*x*_ MXene, 1.0 g of Ti_3_C_2_T_*x*_ powder was mixed with 2 mL of tetramethyl ammonium hydroxide (TMAOH) and manually shaken for 3 minutes. This produced a deeper black color in the MXene wet powder. 20 mL of deionized water (DI water) was added to the black Ti_3_C_2_T_*x*_ MXene mixture, and the mixture was stirred for 24 hours at 300 rpm. Ti_3_C_2_T_*x*_ MXene was washed three times using centrifugation at 3500 rpm using deionized (DI) water. The washing of the MXene was as follows: the first two washing cycles took 10 minutes, and the last one took 15 minutes at 3500 rpm. After the first washing cycle, the sluggish green solution of Ti_3_C_2_T_*x*_ MXene can be seen to be strong. After the third washing cycle, the basic Ti_3_C_2_T_*x*_ solution's pH was no longer acidic or basic. MXene can be used to make freestanding MXene films by washing it further with vacuum-assisted filtration. MXene was dried in a vacuum oven at 120 °C for 18 hours.

### 2.3 MXene nanocomposite preparation

Erbium nitrate hexahydrate was added to Ti_3_C_2_T_*x*_ MXene, which was denoted in terms of different atomic weight percentages, with three ratios of Er/Ti_3_C_2_T_*x*_ nanocomposites (T_1_ (0.5 : 2), T_2_ (1 : 2), and T_3_ (1.5 : 2)) synthesized using a sonication method. The procedure involved dispersing 40 mg of Ti_3_C_2_T_*x*_ in 40 mL of DI water and 12 mg of salt in 12 mL of DI water (1 : 1 volume ratio for each case). The solutions were stirred on a hot plate for 60 minutes and sonicated for 2 minutes. The MXene solution was carefully added to the salt solution dropwise, followed by sonication for two minutes after being magnetically stirred on a hotplate for 2 hours. The resulting solution was centrifuged at 4500 rpm, filtered using vacuum-assisted filtration, and kept for drying overnight at 45 °C in a vacuum oven. The dried product was characterized further.

## 3 Electrochemical measurements

For the fabrication of the electrode, 0.8 mg of active mass, *e.g.*, Er@Ti_3_C_2_T_*x*_, Ti_3_C_2_T_*x*_ powders, acetylene black as a conductive agent, 35 mL of Nafion (C_7_HF_13_O_5_S C_2_F_4_) binder with 5% concentration, and 0.1 mg of carbon black were dissolved in 100 μl of ethanol solvent in an 80 : 10 : 10 weight ratio. The mixture solution was kept for sonication for 10 minutes to create a homogeneous ink/slurry. The ink/slurry was then applied dropwise onto a conductive nickel foam (1.5 × 1 cm) and dried at 40 °C in a vacuum oven overnight. The resulting electrode was then pressed at 500 psi for 10 seconds. Before casting the slurry on the nickel foam (the efficiency of water electrolysis can be significantly improved by modifying nickel foam),^40^ the foam was washed by sonicating it in DI water and ethanol for 10 minutes each and then dried on a hot plate. This process ensured that the foam was clean and ready for slurry application.

## 4 Material characterizations

The XRD (Model: Dron 8) was employed to examine crystal structure and phase identification throughout the angular range of 2*θ* from 4° to 80°. Diffraction patterns were acquired for all materials using Cu-Kα wavelength light (*λ* = 1.5406 Å). Scherrer's formula (*D* = *Kλβ* cos *θ*) measures particle size by broadening the diffraction peak. ‘*D*’ indicates crystallite size, *K*′ shows the coefficient (typically 0.89), ‘*λ*’ represents X-ray radiation wavelength, ‘*β*’ represents FWHM, and ‘*θ*’ is the diffraction angle. The surface morphology of the synthesized MXene and nanocomposites was investigated using SEM (JEOL JSM 6490) at 10 kV, while EDX was employed to determine the elemental composition. The chemical bondings of the synthesized materials were analyzed using FTIR (ATR ALPHA). Using RAMAN (uRAMAN 532 TEC-Ci), the internal structural vibrational modes of MXene and nanocomposites containing MXene were investigated.

## 5 Results and discussions

**Fig. 2:**
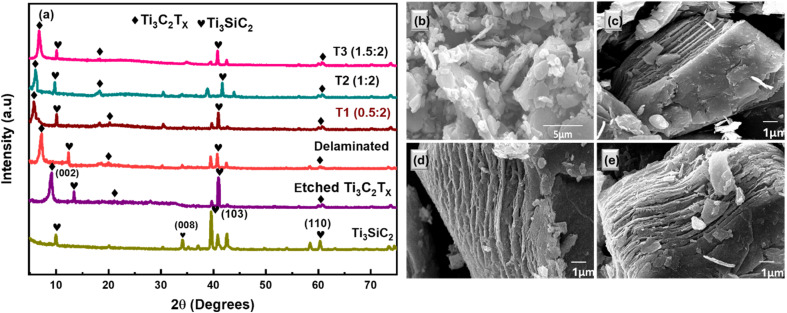
(a) XRD pattern of MAX Ti_3_SiC_2_ (green), etched MXene Ti_3_C_2_T_*x*_ (purple), delaminated MXene (red), and T_1_, T_2_, T_3_ nanocomposites with the best sample T_1_ (brown), (b) scanning electron microscopy (SEM) images of Ti_3_SiC_2_ MAX phase at 5 μm, (c) etched Ti_3_C_2_T_*x*_ MXene, (d) delaminated MXene at 1 μm, (e) nanocomposite of Er@ Ti_3_C_2_T_*x*_ at 1 μm.

Fig. 2a represents the XRD pattern of MAX phase Ti_3_SiC_2_, pristine Ti_3_C_2_T_*x*_ MXene, Delaminated MXene, and Er@Ti_3_C_2_T_*x*_ nanocomposites with varying proportions. The crystal planes (002) and (103) of MAX phase Ti_3_SiC_2_ were identified at 9.95° and 39.6°, respectively. The MAX phase-derived MXene was prepared through the wet chemical etching method. Though Ti_3_SiC_2_ treated with HF/H_2_O_2_ mixture showed a shift in (002) peaks to a lower 9.1° angle, yielding Ti_3_C_2_T_*x*_ and an increase in lattice parameter up to 19.4 Å (Fig. 2a purple colour) with *d*-spacing 9.7 Å,^41^ which is mainly attributed to the removal of Si layers, the attachment of surface functional groups introduced during the synthesis route, and the intercalation of adsorbed water molecules between the MXene layers, thereby confirming the successful exfoliation of the MXene sheets.^42^ The XRD analysis of Ti_3_C_2_T_*x*_ MXene, which was synthesized from Ti_3_SiC_2_ confirmed the intercalation of TMAOH between MXene sheets (Fig. 2a, red colour). The intercalation of TMA^+^ ions is indicated by the increase in *d*-spacing from approximately 8.88 Å to 12.18 Å, yielding the shift of the (002) peak from 9.95° to 7.25° angles, respectively. The apparent formation of TiO_2_, as seen by the peak at 25°, indicates that the MXene sample had minimal oxidation, as previously reported.^43,44^ Peaks at 34° indicate the presence of the SiC phase following a successful exfoliation route process. The significant peak at 8.9° degrees on the XRD of MXene corresponds to Ti_3_C_2_T_*x*_, which is shifted to an even lower degree as a result of the delamination process.^45–47^ The attachment of Er^3+^ ions on/to delaminated Ti_3_C_2_T_*x*_ sheets altered the XRD pattern of the sample. Specifically, the (002) peak shifted to about 5.75° angle, which may indicate a change in the crystal structure of the prepared material due to chemical composition (Fig. 2a, brown colour). The corresponding *c*-lattice parameter of the (002) peak increased by 30.7 Å for the T_1_ (0.5 : 2) nanocomposite, and the *d*-spacing by 15.35. This shift indicates that the layer spacings in Ti_3_C_2_T_*x*_ were increased due to the addition of Er.

The morphology and elemental content of the samples have been studied using scanning electron microscopy (SEM) and energy-dispersive X-ray spectroscopy (EDS).^48^Fig. 2b exhibits an unetched bulk Ti_3_SiC_2_ MAX phase. Fig. 2c illustrates an image of etched MAX phase, where a sheet-like structure bears resemblance to the scanning electron microscope (SEM) images presented in prior research on etched Ti_3_C_2_T_*x*_ powder.^49^ The removal of silicon from etched Ti_3_C_2_T_*x*_ MXene results in an extended layered structure, which has been supported by the enhanced *c*-lattice parameter. Fig. 2d shows delaminated Ti_3_C_2_T_*x*_ MXene, TMA^+^ ion intercalation caused MXene layers to expand significantly, indicating their accordion-like morphology.^50^ Additionally, Fig. 2e shows that tiny particles of Er or Er^3+^ ions can be observed on/in between layers or on the surface of the T_1_ (0.5 : 2) nanocomposite of Er@Ti_3_C_2_T_*x*_. As the amount of Er@Ti_3_C_2_T_*x*_ in the system evolves, the rate of reaction slows down. The increased concentration, as observed in the SEM micrographs in Fig. 2e, covers the 2D sheet structure, which may lead to blockages and a reduction in activity.

**Fig. 3:**
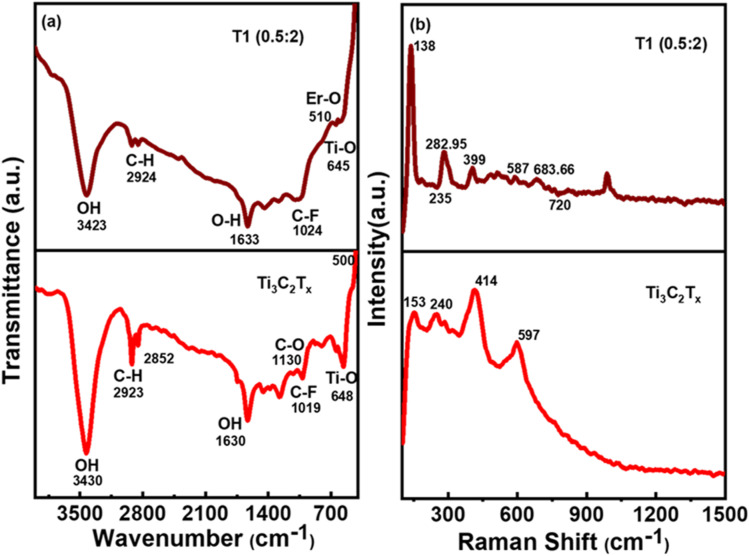
(a) FTIR spectra of delaminated Ti_3_C_2_T_*x*_ MXene (red) and T_1_ nanocomposite (brown), (b) RAMAN spectra of delaminated Ti_3_C_2_T_*x*_ MXene and T_1_ (0.5 : 2) nanocomposite.

The Fourier-transform infrared radiation FTIR spectra of Ti_3_C_2_T_*x*_ MXene and Er@ Ti_3_C_2_T_*x*_ or T1 (0.5 : 1) nanocomposite have been shown in Fig. 3a. FTIR of all the samples was recorded in the range of 400 to 4000 cm^−1^. FTIR spectra are mostly divided into two regions: the functional group region (4000–1200 cm^−1^) and the fingerprint region (<1200 cm^−1^). The detected functional groups may originate from moisture and environmental influences, while the peaks in the fingerprint region are attributed to Ti–O, Er–O, and functional group stretching vibrations, whose detailed configurations are described as follows.^51,52^ In FTIR spectra, broad absorption bands observed at 3430 cm^−1^ show the vibrational stretching of a hydroxyl group (–OH), which shows the absorbance of water^53^ and peak at 2923 cm^−1^ corresponds to the C–H bond (Fig. 3a red). The O–H vibrational mode is represented around 1630 cm^−1^, and the bands in the fingerprint region correspond to the 1019 cm^−1^ C–F bond and 648 cm^−1^ is associated with the Ti–O stretching band, respectively. The peak at 648 cm^−1^ represents the Ti–O bond, indicating the deformation vibration of Ti and O atoms.^54^ The presence of an oxygen-terminated functional group is confirmed by the C–O bond peak at 1130 cm^−1^. The peaks at 2924 and 1451 cm^−1^ were assigned to –CH_2_ and CH, respectively. Similarly, (Fig. 3a-brown) illustrates the FTIR spectra of T_1_ (0.5 : 1) nanocomposite, where absorption bands at 3423 cm^−1^, 2924 cm^−1^, 2850 cm^−1^, 1633 cm^−1^, 1024 cm^−1^ and 510 cm^−1^ can be observed. At 3423 cm^−1^, the broad band corresponds to the –OH vibrational stretching. The bands at 2924 cm^−1^ and 2850 cm^−1^ correspond to the C–H vibrational stretching vibrations. While the peak at 1633 cm^−1^ represents O–H bending vibrations. The bands at 1024 cm^−1^ and 645 cm^−1^ are relevant to the C–F and Ti–O stretching modes, respectively.^54^ As reactive sites of Ti_3_C_2_T_*x*_ functionalization, the functional groups can function as highly activated sites. Additionally, there is a prominent peak of Er–O characteristic stretching vibration at 510 cm^−1^ caused by the stretching vibration. No new peaks have been seen in the FTIR spectrum of Er@Ti_3_C_2_T_*x*_. As the peaks for Ti_3_C_2_T_*x*_'s functional terminations diminish, it's clear that Er has grown successfully on Ti_3_C_2_T_*x*_.

The Raman spectra of delaminated Ti_3_C_2_T_*x*_ MXene powder and Er@Ti_3_C_2_T_*x*_ or T_1_ (0.5 : 1) nanocomposite are shown in Fig. 3b. The observed presence of Raman-active phonon modes at various vibrational frequencies indicates the existence of functional groups on the surface of MXene. Raman spectroscopy was used to study structure, phase shift, crystallinity, and molecular interface interaction. Four phonon peaks of the crystal structure at 153, 240, 414, and 597 cm^−1^ have been observed in the Raman spectrum of Ti_3_C_2_T_*x*_ (Fig. 3b red). The spectrum of Ti_3_C_2_T_*x*_ matches that reported in the previous literature.^55^ The spectral modes at 240 cm^−1^ and 597 cm^−1^ are attributed to out-of-plane stretching vibrations of Ti_2_ and C, respectively^56^ utilized to study the intercalation mechanism between sheets or the adsorption occurring on the surface of MXene. The Raman bands between 300–800 cm^−1^ are particularly associated with the Ti–C bond in MXene.^57^ The subsequent vibration contains surface groups, Si, and C atomic planes. In addition, the Ti_2_ atoms in-plane and out-of-plane vibrations of the outer layer, also attached with functional groups and carbon atoms, are identified at 282.95 cm^−1^ and 399 cm^−1^, respectively. It is related to the phonon modes E_g_ (Ti, C, O) and A_1g_ (Ti, C, O). Sharper Raman peaks, indicative of increased crystallinity, were observed for MXene and T1 composite.^58^ Observation of the phonon peak at 587 cm^−1^ confirms the E_g_ and A_1g_ vibrations of C atoms (Fig. 3b brown). Adjacent C-atomic planes form longitudinal modes at 683.66 cm per phonon frequencies, which correspond to A_g_ modes^46,59^ peak shifting in position is observed when both datasets are compared. The upshifting of the peak's position is attributed to the elongation of bonds. The structural atomic strain is also linked to the prolongation of bonds. In Fig. 3b, the Raman data also show the M–T_*x*_ bond stretching or weakening due to the shielding effect of the Er atom as it attaches to the surface. The Raman spectra of MXene can be broadly classified into four regions, Raman laser has a resonant peak of about 138 cm^−1^. The MXene flakes have mainly E_g_ (in-plane) and A_1g_ (out-of-plane) vibrational modes associated with titanium carbide atoms in the outer layer, Ti atoms, C atoms, and functional group atoms. Most of the atoms within the MXene unit cell actively participate in these vibrations, rendering them the most rigid of all potential vibrations. Surface functional groups that attach to the titanium atom cause vibrations in the adjacent region. E_g_ and A_1g_ (in- and out-of-plane) carbon atom vibrations occurred in the fourth range from 570 to 720 cm^−1^.^60^

### 5.1 Electrochemical performance analysis

On a Gamry Interface 1010B potentiostat with a three-electrode system ^61–63^ in a 1 M KOH (pH = 14) electrolyte, electrochemical testing was conducted. A functional electrode was used to evaluate the as-synthesized MXene and its composites with variable ratios. Ni foam served as the current collector for the active electrode, allowing fast mass and electron transfer, while a platinum wire and an Ag/AgCl (3.5 M KCl) electrode served as counter and reference electrodes, respectively. The polarization curves' Tafel slope and overpotential indicate activity. Changes in the overpotential or current as a function of time are used to characterize stability. The Nernst equation, *E*_RHE_ = *E*_Ag/AgCl_ + 0.0591 pH + 0.1976, was used to analyze all potentials in LSV in reversible hydrogen electrode (RHE), and both the OER and the HER were studied at a fixed low scan rate of 10 mV s^−1^.^62,63^ To determine the kinetic efficiency of the prepared catalyst, the Tafel slope is computed using the equation *η* = *a* + *b* log *j*, where *b* represents the Tafel slope and *η* denotes the overpotential. Composites of Ti_3_C_2_T_*x*_ were prepared with Er in different mass ratios, *i*.*e.*, 0.5 : 2, 1 : 2 : 2, and 1.5 : 2. MXene and Er: MXene samples were synthesized and labelled as T_1_, T_2_, T_3_, respectively. Among all these ratios, Er@Ti_3_C_2_T_*x*_ (0.5 : 2), denoted as T_1_ gave the best results for overall water splitting applications. The EIS measurement was taken by a sinusoidal voltage signal of 10 mV and a frequency range of 20 kHz to 0.1 Hz. The chronoamperometry test was used for long-term durability testing at 0.6 V constant voltage.

### 5.2 Water electrolysis electrochemistry

Although electrocatalytic water splitting is the typical approach for hydrogen production, it only accounts for 4% of overall hydrogen production.^64,65^

Water electrolysis (reduction and oxidation)4
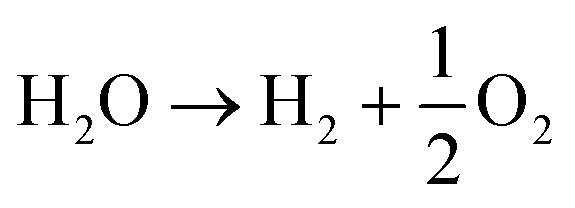


Involves cathodic (HER) and anodic (OER) half-cell reactions, yielding H_2_ and O_2_ (respectively).^66^

Cathode: the hydrogen evolution process (HER) entails the reduction of protons (H^+^), resulting in the formation of hydrogen gas (H_2_).

5 2H^+^ + 2e^−^ → H_2_

Anode: in the Oxygen Evolution Reaction (OER), water molecules (H_2_O) undergo oxidation,^67^ resulting in the production of oxygen gas (O_2_).

6 2H_2_O → O_2_ + 4H^+^ + 4e^−^

For determining overpotential to standard 10 mA cm^−2^ (*η*@10 mA cm^−2^), a standard diagram of current density *versus* voltage is utilized. This involves measuring the generated current with the provided voltage using a potentiostat. The resultant current density is then determined by dividing the current by the catalyst's surface area on the electrode. Typically, the oxygen evolution reaction commences at the end of the non-faradaic zone, at which point the onset potential is identified. A lowered onset potential indicates that an OER catalyst is better. At each electrode, the following are the usual half-cell reactions:

### 5.3 Linear sweep voltammetry (LSV)

#### 5.3.1 Oxygen evolution reaction (OER)

In alkaline/aqueous electrolyte

7 4OH^−^ → O_2_ (g) + 2H_2_O (l) + 4e^−^

**Fig. 4:**
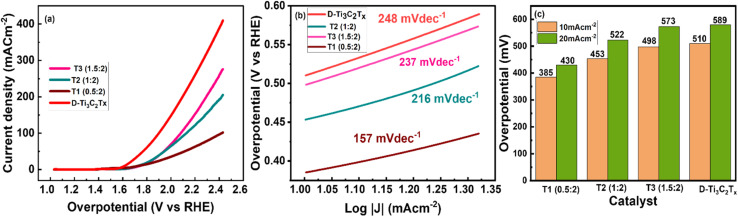
(a) Polarization curve of OER against RHE, (b) Tafel slope of delaminated MXene Ti_3_C_2_T_*x*_ and T_1_ nanocomposite, (c) comparison of the current density at 10 mA cm^−2^ (*η*_10_) and 20 mA cm^−2^ (*η*_20_).

Using LSV polarization curves (electrochemical system to V *versus* RHE stimuli), Fig. 4a depicts the response of delaminated MXene Ti_3_C_2_T_*x*_ and Er@Ti_3_C_2_T_*x*_ nanocomposites (T_1_, T_2_, and T_3_) for OER activity at potential varied from 0 to 1.4 V at a scan rate of 10 mv s^−1^ in 1 molar KOH electrolyte.^68–71^ The T_1_ (0.5 : 2) nanocomposite exhibited the highest activity, the lowest onset potential, the lowest overpotential *η*_10_ (385 mV), and the least Tafel slope of 157 mV dec^−1^. The other electrocatalysts Ti_3_C_2_T_*x*_, T_2_ (1 : 2), and T_3_ (1.5 : 2) showed overpotentials of 510 mV, 498 mV, and 453 mV, respectively, at the same current density of 10 mA cm^−2^ and Tafel slopes of 248 mV dec^−1^, 237 mV dec^−1^, and 216 mV dec^−1^. The Tafel slope of all materials is depicted in Fig. 4b. Moreover, the OER reaction of electrocatalytic kinetics was engaged by Tafel slope (log(|*j*|) *versus* over-potential (*η*)) in Fig. 4c. Increased Er salt concentration causes a decrease in OER activity, which may be associated with active site overloading and blockage of ion transform and active sites. The employment of a minimal quantity of intercalated erbium as a redox species can yield exceptional performance in linear sweep voltammetry for hydrogen and oxygen evolution reactions. This approach effectively balances the necessity for a robust signal with the prevention of mass transport limitations.

#### 5.3.2 Hydrogen evolution reaction (HER)

**Fig. 5:**
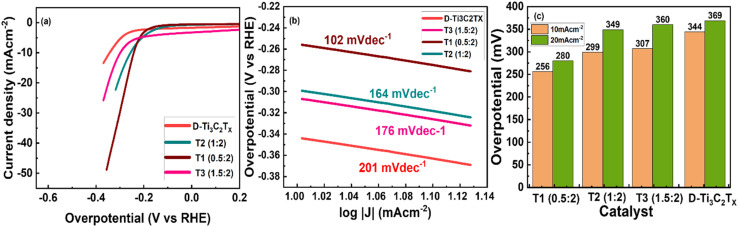
(a) Polarization curve of HER against RHE, (b) Tafel slope of delaminated MXene Ti_3_C_2_T_*x*_ and T_1_ nanocomposite, (c) comparison of the current density at 10 mA cm^−2^ (*η*_10_) and 20 mA cm^−2^ (*η*_20_).

The process of the hydrogen evolution reaction (HER) generally occurs in alkaline electrolytes. It typically begins with a phase where water dissociates and forms a proton (H^+^) adsorbed on the surface of the electrode, which is known as the Volmer step (Fig. 5a).^69^ Then, it was followed by the Tafel step, where adsorbed species recombine or absorbance of water molecules with hydrogen in a coupling, and the Heyrovsky step, followed by the electron. The electrochemical desorption occurred, and it created molecular hydrogen at the electrode surface M as a result of the Heyrovsky step.^68^ The T_1_ nanocomposite follows the Volmer–Heyrovsky pathway for the HER process during the determining step (RDS) according to the literature^72,73^ and is indicated by its slope value. The reaction is followed as:H_2_O + M + e^−^ → MH_ads_ + OH^−^ Volmer step: 120 mV dec^−1^MH_ads_ + H_2_O + e^−^ → H_2_ + M + OH^−^ Heyrovsky step: 40 mV dec^−1^

Tafel slope values are represented in Fig. 5b and the values T_1_, T_2_, and T_3_ observed were 102, 164, and 176 mV dec^−1^, respectively. Whereas T1 has 102 mV dec^−1^, which is the lowest. Furthermore, the Tafel slopes of pure Ti_3_C_2_T_*x*_ are 201 mV dec^−1^, respectively. Using the nickel foam substrate caused a significant enhancement in the overall performance of the T_1_ nanocomposite, which could be due to Er's strong interaction with Ti_3_C_2_T_*x*_ on a nickel foam substrate. The nickel substrate in the composite structure acts as a conductive support and facilitates a fast transfer channel for electron mass. Additionally, the nickel foam has demonstrated very low activity for OER and HER, indicating that it has a negligible impact on the entire water splitting process. The flow of electrolytic ions into the active sites of the sample by the phenomenon of creating a narrow path could be stimulated by using the porous nickel materials. Additionally, the robust bonding of Er onto MXene nanosheets, coupled with Er's active sites, has resulted in improved electrochemical performance. XRD analysis showed that the intercalation of Er into MXene layers facilitated faster ion diffusion pathways. The interaction between the surface oxygen at Ti_3_C_2_T_*x*_ and H is quite strong, which impedes the escape of H_2_ gas. This interaction also enhanced the capability of Ti_3_C_2_T_*x*_ and expedited the charge transfer process. Fig. 5c shows the overpotential exhibited by all studied materials.

### 5.4 EIS analysis

**Fig. 6:**
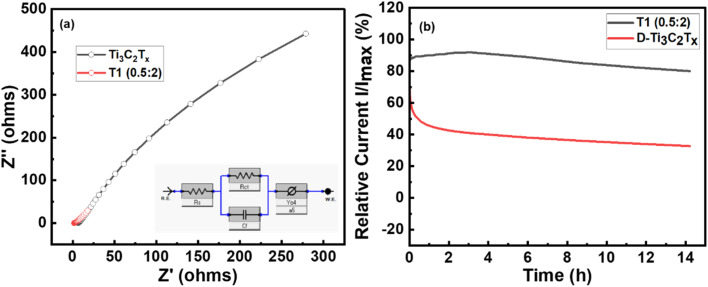
(a) EIS curves of delaminated Ti_3_C_2_T_*x*_ MXene and Er intercalated Ti_3_C_2_T_*x*_ or T_1_ (0.5 : 2) nanocomposite along with an inset equivalent circuit. (b) Chronoamperometry curves of delaminated Ti_3_C_2_T_*x*_ MXene and Er intercalated Ti_3_C_2_T_*x*_ or T1 (0.5 : 2) for 14 h.

The process involving electron transport was investigated using electrochemical impedance spectroscopy (EIS), and the frequency range of the EIS ranged from 0.1 kHz to 20 kHz. The EIS patterns of the delaminated Ti_3_C_2_T_*x*_ and Er@Ti_3_C_2_T_*x*_/T1 (0.5 : 2) composite structures and equivalent circuit model are shown in Fig. 6a. A basic Randle model incorporating a constant phase element was employed in series, where the electrolyte solution between the cell's electrodes is responsible for the solution resistance (*R*_s_), the electrolyte interface's charge transfer resistance (*R*_ct_), which refers to polarization resistance, and faradaic capacitance (*C*_dl_) are displayed in Fig. 6a following fitting for the EIS results.^74^ A structure containing Ti_3_C_2_T_*x*_ had the lowest electron transfer resistance (*R*_ct_) at 342.7 ohms, followed by an Er@Ti_3_C_2_T_*x*_ (T_1_) structure with 77.61 ohms and solution resistance or ohmic resistance 6.731 and 1.69 ohms, respectively. The results of the Nyquist plot show Ti_3_C_2_T_*x*_ MXene has enhanced conductivity and quicker transfer mechanisms, resulting in faster reaction kinetics.

### 5.5 Chronoamperometry

Maintaining the stability of electrochemical materials is a critical factor for their commercial applications. Fig. 6b represents the stability of pristine Ti_3_C_2_T_*x*_ MXene and T_1_ composite sample by a chronoamperometry test was performed at 0.6 V in a 1 M KOH solution. The relative current stability of 80% and 34% was observed after 14 hours of operation for T_1_ nanocomposite and delaminated Ti_3_C_2_T_*x*_ MXene, respectively, which indicates the stable behaviour attributed to the material's structural features.^75^ During chronoamperometry testing, ion diffusion resistance was observed to reduce at the interface of electrode–electrolyte, which facilitated the penetration of ions *via* the active sites in the prepared material due to the electrochemical activation process.

### 5.6 Mechanistic role of Er intercalation on enhancing HER/OER activity

The incorporation of erbium (Er) into Ti_3_C_2_T_*x*_ MXene significantly enhances its electrocatalytic performance for hydrogen and oxygen evolution reactions.^76,77^ Erbium, with its partially filled 4f orbitals, interacts strongly with the Ti sites and surface terminations of MXene, leading to modulation of the electronic structure and optimization of the adsorption energies of reaction intermediates.^78^ This adjustment lowers the kinetic barriers associated with *H adsorption in HER and *OH/OOH formation in OER.^79,80^ In alkaline media, the strong interaction between Er and Ti_3_C_2_T_*x*_ facilitates water dissociation by reducing the energy barrier for the Volmer step, which is generally rate-limiting in HER. Furthermore, Er sites preferentially stabilize *OH intermediates, while Ti sites favor *H adsorption, resulting in a synergistic effect that improves the kinetics of both half-reactions.^81^ Structurally, Er intercalation increases the interlayer spacing of MXene, suppresses restacking, enhances electrolyte penetration and ion transport, and the strong Er–MXene interaction induces charge redistribution and enhances electrical conductivity, which directly lowers the kinetic barrier for water dissociation in alkaline HER, and stabilizes oxygenated intermediates during OER.^82^ Together, these factors account for the observed improvements in catalytic activity and durability of Er-intercalated Ti_3_C_2_T_*x*_ MXene during overall water splitting in alkaline conditions.

## 6 Conclusion

In conclusion, the bifunctional performance of a novel nanocomposite structure in an alkaline electrolyte was enhanced by the deposition of Er onto 2D Ti_3_C_2_T_*x*_ MXene nanosheets for overall water-splitting application. A bare MXene catalyst was prepared and characterized for comparison. In comparison to pure MXene, Er@Ti_3_C_2_T_*x*_ nanocomposites demonstrated exceptional HER activity and OER performance. This improved performance may be attributed to the uniform networking of Er over the surface and the high conductivity of MXene sheets, which increases the ability for excellent, rapid charge transfer to the active sites and an effective synergistic impact for both the oxygen evolution reaction (OER) and the hydrogen evolution reaction (HER), and prevents aggregation. They also contain numerous active sites. Er-based nanocomposites have remarkable durability in alkaline environments, leading to the exceptional stability of the nanostructure. This research implies that the Er@Ti_3_C_2_T_*x*_ nanocomposite might replace commercial noble metal electrocatalysts due to its efficient cost and eco-friendliness. Furthermore, the present study provides potential for the development of nanocomposites based on 2D MXene and Er to facilitate the efficient bifunctional electrocatalysis required for the overall water splitting process.

## Author contributions

Shamaila Fatima managed the entire experimentation and manuscript preparation process; Irfan Ali contributed to data analysis and proofreading; Aumber Abbas and Azhar Ali Haidry contributed to data collection, while Syed Rizwan supervised the project, assisted with manuscript writing, and conceived the research idea.

## Conflicts of interest

The authors declared there is no competing interest.

## Supplementary Material

RA-015-D5RA05111H-s001

## Data Availability

The data related to the work is presented within the manuscript and other supporting data will be made on request. Supplementary information is available. See DOI: https://doi.org/10.1039/d5ra05111h.
