# Supplementary material for: Er-intercalated Ti3C2Tx MXene electrocatalyst for efficient energy conversion
Source: RSC Adv. 2025 Oct 8;15(44):37379–90. doi: 10.1039/d5ra05111h (PMC12506646; doi:10.1039/d5ra05111h)
Supplement: RA-015-D5RA05111H-s001 [file RA-015-D5RA05111H-s001.pdf]

Supplementary Information (SI) for RSC Advances.
This journal is © The Royal Society of Chemistry 2025

Er-Intercalated $Ti_3C_2T_x$ MXene Electrocatalyst for Efficient Energy Conversion

Shamaila Fatima,[a] Irfan Ali,[a] Aumber Abbas,[b] Azhar Ali Haidry,[c] Syed Rizwan[a]

[a] Physics Characterization and Simulations Lab (PCSL), Department of Physics & Astronomy, School of Natural Sciences (SNS), National University of Sciences and Technology (NUST), Islamabad 44000, Pakistan.

[b] School of Materials Engineering, Jiangsu University of Technology, Changzhou 213001, China.

[c] College of Materials Science and Technology (MST), Nanjing University of Aeronautics and Astronautics (NUAA), JiangJun Avenue 29, Nanjing 21101, PR China.

Corresponding author: Syed Rizwan: syedrizwan@sns.nust.edu.pk, syedrizwanh83@gmail.com; Tel: +92 51 886 5599.

The elemental composition of 2D MXene nanosheets was determined by energy dispersive X-ray spectroscopy (EDX). Fig. 2f shows the EDS results that the weight percentage of multilayered MXene Ti_3C_2Tx and nanocomposite T1(0.5:2) contains elemental peaks of Er, Ti, Si, C, O, and F. In EDX, Si and C confirm its SiC phase and that Si remains after the $Ti_3C_2T_x$ MXene phase. Furthermore, the

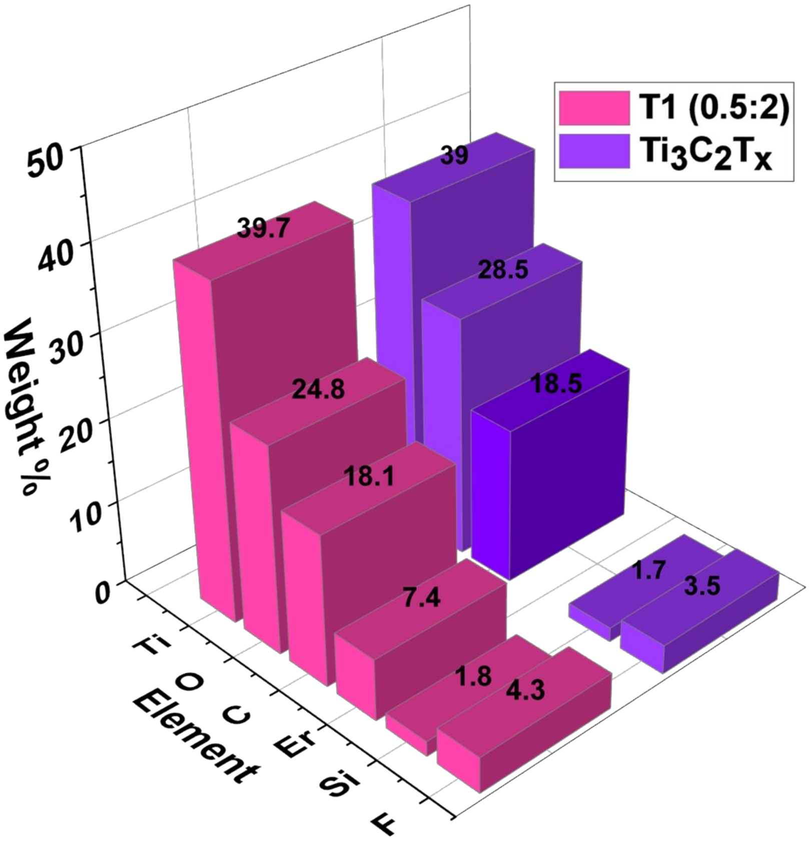

effective synthesis of the sample is confirmed by the presence of Er in the T1 (0.5:2) nanocomposite.

Supplementary figure 1 X-ray spectroscopy (EDX) *(a) weight percentage EDX spectra of delaminated MXene and Er@$Ti_3C_2T_x$, respectively.*

Additionally, Table 1 presents the reported HER and OER activities of synthesized MXenes and their composites in both acidic and alkaline media.

Table 1 *Overview of (HER & OER) electrocatalytic activities of MXene-based ($Ti_3C_2T_x$) electrocatalysts.*

**Catalyst**	**Electrolyte**	**Activity**	**Overpotential @ 10 mA cm^{-2} (η_{10} mV)**	**Reference**
Co^{3+}-Ti_2CT_x	1.0 M KOH	OER	420	[1]
Co/N-CNTs@$Ti_3C_2T_x$	0.1 M KOH	OER	411	[2]
FeNi@GO/$Ti_3C_2T_x$	1.0 M KOH	OER	370	[3]
1T/2H $MoSe_2$/MXene	1.0 M KOH	OER	340	[4]
$Ni_{1.5}Co_{1.5}(PO_4)_2$@$Ti_3C_2T_x$	1.0 M KOH	OER	286	[5]
NiFeMo-$Ti_3C_2T_x$	1.0 M KOH	OER	280	[6]
CeCoFePLDH-$Ti_3C_2T_x$	1.0 M KOH	OER	266	[7]
FeNi@GO-$Ti_3C_2T_x$	1.0 M KOH	HER	470	[3]
Co^{+3}-Ti_2CT_x	1.0 M KOH	HER	458	[1]
Zn/$Ti_3C_2T_x$	0.5M H_2SO_4		422	[8]
Graphitic-C_3N_4/ $Ti_3C_2T_x$ film	0.1 M KOH	HER	420	[9]
Fe_2B/$Ti_3C_2T_x$	0.1 M KOH	HER	294	[10]
MoS_2/$Ti_3C_2T_x$	0.5 M H_2SO_4	HER	280	[11]
Cl-MXene ($Ti_3C_2Cl_2$)	1.0 M KOH	HER	259	[12]
Pt NP/$Ti_3C_2T_{x\,x}$	0.5 M H_2SO_4	HER	226	[13]
NiSe2/Ti3C2Tx	0.5 M H_2SO_4	HER	200	[14]
Er@$Ti_3C_2T_x$	1.0 M KOH	HER	256	**This work**
Er@$Ti_3C_2T_x$	1.0 M KOH	OER	385	**This work**

Reference

[1] S.Y. Pang, Y.T. Wong, S. Yuan, Y. Liu, M.K. Tsang, Z. Yang, H. Huang, W.T. Wong, J. Hao, Universal strategy for HF-free facile and rapid synthesis of two-dimensional MXenes as

multifunctional energy materials, ACS PublicationsSY Pang, YT Wong, S Yuan, Y Liu, MK Tsang, Z Yang, H Huang, WT Wong, J HaoJournal of the American Chemical Society, 2019•ACS Publications 141 (2019) 9610–9616. https://doi.org/10.1021/JACS.9B02578.

[2] Y. Zhang, H. Jiang, Y. Lin, H. Liu, Q. He, C. Wu, T. Duan, L. Song, In Situ Growth of Cobalt Nanoparticles Encapsulated Nitrogen-Doped Carbon Nanotubes among Ti3C2Tx (MXene) Matrix for Oxygen Reduction and Evolution, Wiley Online LibraryY Zhang, H Jiang, Y Lin, H Liu, Q He, C Wu, T Duan, L SongAdvanced Materials Interfaces, 2018•Wiley Online Library 5 (2018). https://doi.org/10.1002/ADMI.201800392.

[3] S.K. Raj, G.R. Bhadu, P. Upadhyay, V. Kulshrestha, Three-dimensional Ni/Fe doped graphene oxide@ MXene architecture as an efficient water splitting electrocatalyst, ElsevierSK Raj, GR Bhadu, P Upadhyay, V KulshresthaInternational Journal of Hydrogen Energy, 2022•Elsevier 47 (2022) 41772–41782. https://doi.org/10.1016/J.IJHYDENE.2022.05.204.

[4] N. Li, Y. Zhang, M. Jia, X. Lv, X. Li, R. Li, … X.D.-E., undefined 2019, 1T/2H MoSe2-on-MXene heterostructure as bifunctional electrocatalyst for efficient overall water splitting, ElsevierN Li, Y Zhang, M Jia, X Lv, X Li, R Li, X Ding, YZ Zheng, X TaoElectrochimica Acta, 2019•Elsevier (n.d.).

[5] R.S. Mane, D. Zaroliwalla, G. Periyasamy, N. Jha, Leafy ZIF-Derived Bi-Metallic Phosphate-Mxene Nanocomposites for Overall Water Splitting, Wiley Online LibraryRS Mane, D Zaroliwalla, G Periyasamy, N JhaSmall, 2025•Wiley Online Library 21 (2025). https://doi.org/10.1002/SMLL.202503228.

[6] S. Pal, E. Chaturvedi, C. Das, N. Sinha, T. Ahmed, P.R.- Nanoscale, undefined 2025, NiFeMo layered triple hydroxide and MXene heterostructure for boosted oxygen evolution reaction in anion exchange membrane water electrolysis, Pubs.Rsc.OrgS Pal, E Chaturvedi, C Das, N Sinha, T Ahmed, P RoyNanoscale, 2025•pubs.Rsc.Org (n.d.). https://pubs.rsc.org/en/content/articlehtml/2025/nr/d5nr00419e (accessed September 11, 2025).

[7] Y. Pan, S. Perumal, M. Sakthivel, … M.Y.-A. at S., Vanadium Carbide Mxene Supported Cerium Based Trimetallic Phosphide as an Efficient Electrocatalyst for Alkaline Water Splitting, Papers.Ssrn.Com (n.d.). https://papers.ssrn.com/sol3/papers.cfm?abstract_id=5059133 (accessed September 11, 2025).

[8] B. Saini, K. Harikrishna, D. Laishram, R. Krishnapriya, R. Singhal, R.K. Sharma, Role of ZnO in ZnO Nanoflake/Ti3C2MXene Composites in Photocatalytic and Electrocatalytic Hydrogen Evolution, ACS Appl Nano Mater 5 (2022) 9319–9333. https://doi.org/10.1021/ACSANM.2C01639/ASSET/IMAGES/LARGE/AN2C01639_0010.JPEG.

[9] T.Y. Ma, J.L. Cao, M. Jaroniec, S.Z. Qiao, Interacting carbon nitride and titanium carbide nanosheets for high-performance oxygen evolution, Wiley Online LibraryTY Ma, JL Cao, M Jaroniec, SZ QiaoAngewandte Chemie International Edition, 2016•Wiley Online Library 55 (2016) 1138–1142. https://doi.org/10.1002/ANIE.201509758.

[10] S. Chauhan, K. Joshi, P. Pataniya, P.S.-R. Energy, undefined 2025, Fe2B/MXene@ NF electrocatalyst for efficient water splitting and green hydrogen production at high current densities, ElsevierSV Chauhan, KK Joshi, PM Pataniya, P Sahatiya, G Bhadu, CK SumeshRenewable Energy, 2025•Elsevier (n.d.). https://www.sciencedirect.com/science/article/pii/S0960148125000321?casa_token=ra0_Bu

4wiDIAAAAA:wY_FeFXV19lND5t6ECAped8tMCiY8M9MJkvwh8NV61IZqD-9sOyGn-56DQTGNyfrk5lbBBG2AaA (accessed September 11, 2025).

[11] L. Huang, L. Ai, M. Wang, J. Jiang, S.W.-I.J. of, undefined 2019, Hierarchical MoS2 nanosheets integrated Ti3C2 MXenes for electrocatalytic hydrogen evolution, ElsevierL Huang, L Ai, M Wang, J Jiang, S WangInternational Journal of Hydrogen Energy, 2019•Elsevier (n.d.).

[12] B. Sarfraz, M.T. Mehran, M.M. Baig, S.R. Naqvi, A.H. Khoja, F. Shahzad, HF free greener Cl-terminated MXene as novel electrocatalyst for overall water splitting in alkaline media, Wiley Online LibraryB Sarfraz, MT Mehran, MM Baig, SR Naqvi, AH Khoja, F ShahzadInternational Journal of Energy Research, 2022•Wiley Online Library 46 (2022) 10942–10954. https://doi.org/10.1002/ER.7895.

[13] J. Filip, S. Zavahir, L. Lorencova, T. Bertok, A. Bin Yousaf, K.A. Mahmoud, J. Tkac, P. Kasak, Tailoring electrocatalytic properties of Pt nanoparticles grown on Ti3C2Tx MXene surface, Iopscience.Iop.OrgJ Filip, S Zavahir, L Lorencova, T Bertok, AB Yousaf, KA Mahmoud, J Tkac, P KasakJournal of The Electrochemical Society, 2019•iopscience.Iop.Org 166 (2019) H54–H62. https://doi.org/10.1149/2.0991902JES/META.

[14] H. Jiang, Z. Wang, Q. Yang, L. Tan, L. Dong, M. Dong, Ultrathin Ti3C2Tx (MXene) Nanosheet-Wrapped NiSe2 Octahedral Crystal for Enhanced Supercapacitor Performance and Synergetic Electrocatalytic Water, SpringerH Jiang, Z Wang, Q Yang, L Tan, L Dong, M DongNano-Micro Letters, 2019•Springer 11 (2019). https://doi.org/10.1007/S40820-019-0261-5.
